# The Utility of Euroflow MRD Assessment in Real-World Multiple Myeloma Practice

**DOI:** 10.3389/fonc.2022.820605

**Published:** 2022-05-18

**Authors:** Rose Turner, Anna Kalff, Krystal Bergin, Malgorzata Gorniak, Shaun Fleming, Andrew Spencer

**Affiliations:** ^1^ Department of Haematology, Alfred Health, Melbourne, VIC, Australia; ^2^ Department of Haematology, Alfred Health and Monash University, Melbourne, VIC, Australia

**Keywords:** MRD, myeloma, VCD, induction, transplant, ASCT, real-world

## Abstract

Measurable residual disease (MRD) is being recognised as an optimal method for assessing depth of response, identifying higher risk of relapse, and guiding response-based treatment paradigms for multiple myeloma (MM). Although MRD negativity is increasingly replacing complete response as the surrogate endpoint in clinical trials, its role in real-world practice is less established. We retrospectively analyzed EuroFlow MRD results from patients with newly diagnosed MM (NDMM) who underwent bortezomib, cyclophosphamide and dexamethasone (VCD) induction and high dose melphalan conditioned autologous stem cell transplant (ASCT) at the Alfred Hospital between January 2016 and December 2020. Next generation flow MRD evaluation was performed 3 months following ASCT using the standardised EuroFlow platform. 112 patients with available MRD data were identified to have received VCD induction followed by ASCT. Post ASCT MRD was undetectable in 28.6% of patients. Those who achieved MRD negativity had significantly longer progression free survival (PFS) than those with persisting MRD (24-month PFS of 85% [95% CI: 72.4-99.9%] vs 63% [95% CI: 52.9-75.3%], p = 0.022). Maintenance therapy was associated with improved PFS regardless of MRD status (24-month PFS of 100% [95% CI: NA, p = 0.02] vs 73% [95% CI: 53.1-99.6%] in MRD negative, and 75% [95% CI: 64.2-88.6%] vs 36% [95% CI: 20.9-63.2%, p = 0.00015] in MRD positive patients). Results from this retrospective study of real-world practice demonstrate that Euroflow MRD analysis following standard VCD induction and ASCT in NDMM is feasible and allows more accurate prognostication, providing a platform for response adaptive therapies.

## Introduction

In the last 20 years, the treatment landscape for multiple myeloma (MM) has rapidly evolved, with marked improvement in patient outcomes. The introduction of novel agents, use of high-dose chemotherapy-conditioned autologous stem cell transplantation (ASCT), and incorporation of sequential phases of treatment have resulted in increasing depth of response and progression-free survival (PFS) (1–5). Despite unprecedented rates of complete response (CR), most patients ultimately relapse, particularly those with high-risk (HR) cytogenetic abnormalities, despite similar rates of CR as compared with standard-risk (SR) patients. This demonstrates the need for a more sensitive response assessment as well as new response criteria to identify deeper responses than current definitions.

Measurable residual disease (MRD) is increasingly being recognized as a more informative method for assessing depth of response, identifying patients at higher risk of relapse, and therefore potentially guiding response-based treatment paradigms (6). The role of MRD as a prognostic biomarker has now been well established with data demonstrating that deeper remission, as evidenced by the absence of MRD at levels <1 × 10^−5^, correlates with improved PFS and overall survival (OS), regardless of cytogenetic risk or stage of disease, suggesting that the goal of treatment for patients with newly diagnosed (ND) MM should be to induce the deepest remission possible (7–11).

In the clinical trial domain, increasing CR rates and long-term survival have led to the adoption of MRD negativity over conventional CR as a more accurate measure of the depth of response and a surrogate endpoint for PFS and OS. The availability of increasingly sensitive and standardized MRD platforms, as well as harmonized guidelines for MRD measurement and reporting, has mitigated the potential heterogeneity in MRD analysis and helped define the optimal methods for measurement, time points of assessment, and interpretation and reporting of results (12, 13). In particular, the validation and publication of the EuroFlow Next-Generation Flow (NGF) MRD methodology have allowed the widespread use of a rapid, fully standardized, highly sensitive (10^−5–6^), and reproducible MRD assay in laboratories globally (14, 15).

Outside of clinical trials, the role of MRD as a measure of response and a means of prognostication is less established and currently not standard of care. However, there is growing evidence to support risk stratification according to MRD over conventional response criteria. Data on the outcomes of MRD measurement in the real-world setting are significantly lacking, with few publications to date. Similarly, despite its incorporation into the IMWG response criteria, there is currently no evidence that MRD can be used to drive therapeutic choices in standard clinical practice. In Australia, the standard of care for transplant-eligible (TE) newly diagnosed multiple myeloma (NDMM) over the last 5 years has been that of bortezomib-based triplet induction therapy, most commonly in the form of bortezomib, cyclophosphamide, and dexamethasone (VCD), followed by ASCT.

EuroFlow MRD assessment 3 months following ASCT is the current practice for all NDMM patients undergoing standard induction and intensification at our center, the Alfred Hospital, a tertiary hospital and transplant center in Melbourne. We have retrospectively analyzed posttransplant EuroFlow MRD results from patients who underwent VCD induction followed by high-dose melphalan (HDM) conditioned ASCT for the treatment of NDMM over the last 5 years. We present the results of these data herein as one of the few published real-world experiences of MRD assessment and utilization in the standard-of-care treatment of MM patients.

## Methods

Patients undergoing VCD induction and HDM ASCT for NDMM at the Alfred Hospital between January 2016 and December 2020 were identified through a local database search. Clinical data, including disease characteristics, the International Staging System (ISS) stage of disease, cytogenetic abnormalities, treatment specifics, disease response, and time to progression, were retrieved from electronic medical records and pathology results. High-risk cytogenetic abnormalities were defined as deletion 17p, t(4;14), t(14;16), t(14, 20), gain 1q, nonhyperdiploid karyotype, and karyotype deletion (13) *via* standard karyotyping and fluorescence *in situ* hybridization (FISH), with 3 or more cytogenetic abnormalities constituting ultra-high-risk cytogenetics.

Due to patient referral from other state and regional centers, the dosing, route, and schedule of VCD cycles received prior to ASCT varied between patients. Stem cell mobilization was predominantly performed following 3–4 cycles of VCD using granulocyte colony-stimulating factor with the addition of plerixafor in the event of poor mobilization. Pre-ASCT high-dose chemotherapy consisted of 200 mg/m^2^ melphalan. Post-ASCT maintenance approaches likewise varied, consisting predominantly of observation, lenalidomide maintenance (10 mg OD PO, adjusted according to toxicity or renal impairment), or thalidomide consolidation (100 mg PO OD) alone or in combination with prednisolone, dexamethasone, and/or ixazomib (4 mg PO weekly for a maximum of 12 months).

Response assessment was performed 3 months post-ASCT *via* serum and urinary protein electrophoresis, serum-free light chain assay, and bone marrow evaluation and defined according to the International Myeloma Working Group consensus criteria for response (12). NGF MRD evaluation of bone marrow aspirate was performed using the standardized 2-tube 8-color EuroFlow platform (MMMRD panel composition) utilizing a Beckman Coulter Navios flow cytometer and Cytognos Infnicyt™ software (Sanbio BV, Salamanca, Spain). Test sensitivity at a level of 10^−5^ (0.001%) was defined with thresholds for the lower limit of detection (LLOD) of 20 cells in 2 × 10^6^ nucleated BM cells and the lower limit of quantification (LLOQ) of 50 cells in 5 × 10^6^ nucleated BM cells according to the ICCS consensus guidelines (14). “MRD negativity” was defined as the absence of tumor plasma cells within 100,000 bone marrow cells (10^−5^). Patients provided written informed consent in accordance with the Declaration of Helsinki. Survival curves were constructed according to the Kaplan–Meier method, with PFS defined as the time from MRD assessment until disease progression or death of any cause. Differences were tested for statistical significance with the (two-sided) log-rank test, with a *p*-value of <0.05 considered significant. Hazard ratios (HRs; with two-sided 95% CIs) were estimated with a Cox regression model.

## Results

A total of 252 patients underwent ASCT for MM at Alfred Hospital between January 2016 and December 2020. Of these, 112 patients with available MRD data were identified to have received VCD induction followed by HDM ASCT for NDMM. A further 15 patients did not have MRD data available, predominantly due to return to the primary referrer prior to post-ASCT reassessment, and were not included in the final analysis. The majority of patients (96%) received 4-6 cycles of VCD prior to ASCT. ISS stage of disease and cytogenetic risk are reported in **Table 1**.

**Table 1:** Disease characteristics, response, and maintenance therapy.

	Total (*n* = 112)
**ISS stage (*n* (%)**	
I	35 (31)
II	40 (36)
III	23 (21)
Data missing	14 (12)
**Cytogenetic risk (*n* (%)**	
SR	53 (47)
HR	12 (11)
UHR	12(11)
Data missing/failed	35 (31)
**Conventional response (*n* (%)**	
CR	42 (38)
VGPR	31 (28)
PR	34 (30)
<PR	5 (4)
**MRD response (*n* (%)**	
MRD negative	32 (29)
CR	20
VGPR	7
PR	5
MRD negative, suboptimal	8 (7)
MRD positive	72 (64)
**Maintenance therapy (*n* (%)**	
Yes	69 (62)
Lenalidomide	16
Thalidomide (+/− steroid)	32
ITd	19
Other	2
No	39 (35)
Unknown	4 (3)

ISS, International Staging System; HR, high-risk cytogenetic abnormalities (defined as deletion 17p, t(4;14), t(14;16), t(14, 20), gain 1q, nonhyperdiploid karyotype, karyotype deletion (13)); UHR, ultra-high-risk cytogenetic abnormalities (defined as 3 or more cytogenetic abnormalities excluding hyperdiploidy); ITd, ixazomib, thalidomide, and dexamethasone.

At the time of response assessment, 37.5% of patients were in CR, 27.7% in VGPR, 30.3% in PR, and 4.5% in less than PR, including 2 with progressive disease. MRD negativity post-ASCT was seen in 28.6% of patients, including 20 patients in CR, 7 in VGPR, and 5 in PR (**Table 1**). Of those in biochemical PR, 1 patient did not have marrow involvement at diagnosis, and 3 had morphological CR on bone marrow assessment with the persistence of serum paraprotein only. A further 7.1% of patients were MRD negative but with suboptimal specimens due to low total viable nucleated cells; these were included as MRD positive for subsequent PFS analysis. The rate of MRD negativity was seen to increase with increasing cytogenetic risk (SR: 25%, HR: 42%, UHR: 50%, *p* = 0.16) and ISS stage of disease (I: 23%, II: 28%, III: 39%, *p* = 0.4). However, this was not statistically significant.

**Figure 1:**
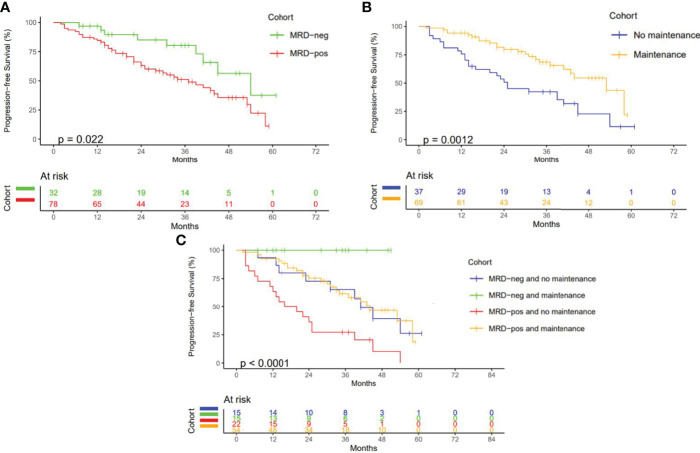
Progression Free Survival **(A)** PFS according to MRD status. **(B)** PFS according to maintenance therapy received. **(C)** PFS according to MRD status and maintenance therapy (months).

After a median follow-up of 38 months, 58 patients (51.8%) had not progressed at the time of the last review, with 24- and 36-month PFS of 69% (95% CI: 60.5%–78.9%) and 59% (95% CI: 49.6%–70%), respectively. Two patients were excluded from PFS analysis due to loss of follow-up, and 2 patients had died without progression of disease. Those who achieved MRD negativity had a significantly longer PFS than those with persisting MRD, with a 24-month PFS of 85% (95% CI: 72.4%–99.9%) versus 63% (95% CI: 52.9%–75.3%, HR = 2.25, 95% CI: 1.09–4.62, *p* = 0.022) (**Figure 1A**). At least 61% of patients had received some form of maintenance therapy following ACST (**Table 1**), with PFS being greater for those who had, with a 24-month PFS of 80% (95% CI: 70.1%–90.8%) versus 51% (95% CI: 36.8%–69.9%, HR = 0.41, 95% CI: 0.23%–0.71%, *p* = 0.0012, **Figure 1B**). Maintenance therapy was found to improve PFS regardless of MRD status, with 24-month PFS increasing from 73% (95% CI: 53.1%–99.6%) to 100% (95% CI: NA, *p* = 0.02) in MRD-negative patients and 36% (95% CI: 20.9%–63.2%) to 75% (95% CI: 64.2%–88.6%, *p* = 0.00015) in MRD-positive patients (**Figure 1C**).

## Discussion

Results from this retrospective study of real-world practice demonstrate that Euroflow MRD analysis following standard VCD induction and ASCT in NDMM is both feasible and informative, allowing greater prognostication as well as the potential for ongoing response adaptive therapy.

This is one of the few studies to report high-sensitivity MRD data for VCD induction, a treatment regimen that continues to be widely used in multiple jurisdictions globally. Published response rates for VCD induction and ASCT have historically been reported according to conventional response criteria without the incorporation of MRD assessment. More recent studies offering deeper response assessment have been limited by small patient sample sizes, the use of conventional multiparameter flow cytometry (MFC) without a stated threshold of sensitivity, and confounding treatment arms (16, 17). Similarly, although multiple studies have compared VCD with other bortezomib-based triplet regimens, given the paucity of MRD data, few have directly compared rates of high sensitivity MRD negativity (18–20).

**Figure 2:**
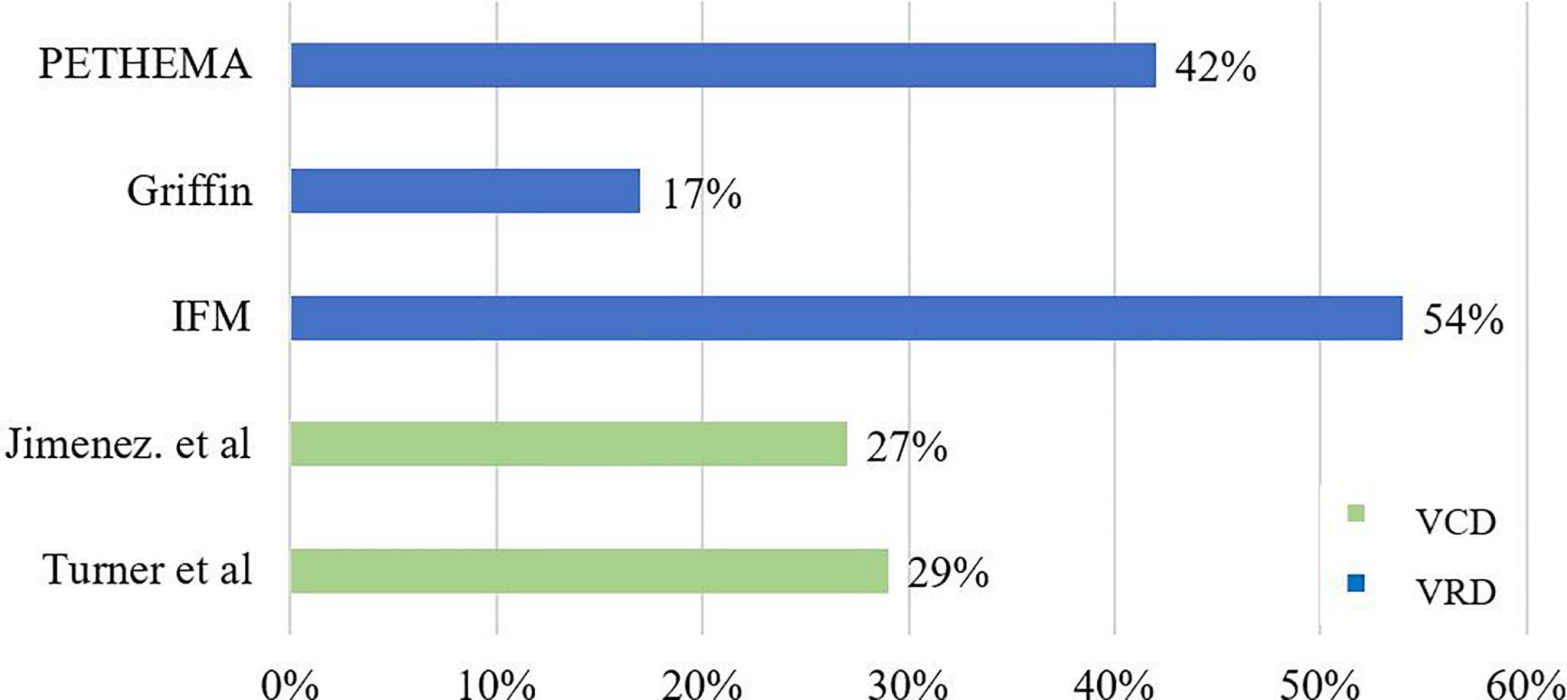
Comparison of MRD Negativity between Clinical Trials.

The reported rates of MRD negativity achieved with bortezomib, lenalidomide, and dexamethasone (VRD) induction and ASCT have varied significantly between studies and dosing regimens (**Figure 2**). The Intergroupe Francophone du Myelome (IFM) trial, one of the first to report MRD following VRD induction in NDMM, found an MRD negativity rate of 54% following 3 cycles of VRD induction and HDM ASCT. However, this study utilized a single 7-color tube MFC assay with a sensitivity of 0.0025%, reported for only 26 patients in a nonintention-to-treat analysis (21). Conversely, the more recent GRIFFIN trial reported an MRD negativity rate of only 16.5% as assessed by next-generation sequencing (NGS, sensitivity of 1 in 10^5^) in 103 NDMM patients in an intent-to-treat-analysis following 4 cycles of VRD induction, ASCT, and 2 cycles of VRD consolidation, with post-ASCT MRD assessment not performed (22). Using a differing treatment regimen again, the PETHEMA/GEM2012MENOS65 trial reported an MRD negativity rate of 42% using EuroFlow NGF MRD assessment following 6 cycles of VRD and ASCT in an intent-to-treat population of 458 NDMM patients (23).

Results from the present study, demonstrating an MRD negativity rate of 28.6% following VCD induction and ASCT, suggest that this regimen compares favorably with VRD and remains an attractive induction option capable of achieving comparable depths of response. Interestingly, the MRD negativity was observed to increase with increasing cytogenetic risk and stage of disease, albeit nonsignificantly, perhaps suggesting an advantage to treating high-risk patients with VCD rather than VRD induction, where MRD negativity is significantly lower in the context of HR versus SR cytogenetics (49% vs. 37%, *p* = 0.04) (11). One of the limitations in the comparison of our data is the lack of an intention to treat analysis, with the exclusion of patients for whom MRD data were unavailable (*n* = 15), as well as the failure to capture patients with primary refractoriness to VCD who went on to receive secondary lines of therapy with or without ASCT.

MRD negativity has been well established to correlate with improved PFS (7–11), an observation confirmed in the present study with a 24-month PFS of 85% for those patients who achieved MRD negativity as opposed to 63% (*p* = 0.022) for those with persisting MRD positivity. Subgroup analysis further demonstrated improved PFS with maintenance therapy, regardless of MRD status, with PFS increasing from 73% to 100% (*p* = 0.02) in MRD-negative patients and from 36% to 75% (*p* < 0.001) in MRD-positive patients, respectively. While limited by the small sample size and confounded by the differing regimens of post-ASCT maintenance therapy, these findings support the utilization of posttransplant MRD testing as well as maintenance therapy in NDMM in suspected VGPR or greater. They also suggest that response adaptive treatment pathways that offer consolidation therapy to deepen response in patients with persisting MRD may improve PFS (24), a hypothesis that is currently being tested in several new response adaptive clinical trials, including the MASTER trial (25). Although the inclusion of a single time point for MRD assessment, currently the standard of care at our center, fails to capture the impact of maintenance therapy on MRD status and loss of MRD negativity over time, further data are needed to establish the benefits of serial MRD assessment in real-world practice. Assessment of MRD based on bone marrow aspirate at a single site likewise raises the theoretical possibility of missing spatially heterogeneous disease and emphasizes the need for more global MRD assays to be incorporated into standard practice (26).

This is one of the few studies to report on MRD analysis in real-world MM practice, with most data to date limited to the clinical trial setting. While analyzing data from real-world practice is challenging due to heterogeneities in patient characteristics, treatment regimens, and response assessment, as well as difficulties inherent to the long-term follow-up of regionally referred patients, such data still allow meaningful inferences to be made and remain essential for guiding treatment practices and improving outcomes. Despite these limitations, this study demonstrates that MRD analysis poststandard induction and ASCT is a feasible and informative time point for deeper response assessment in the real-world setting, allowing for greater prognosis and paving the way for personalized treatment regimens.

## Data Availability Statement

The raw data supporting the conclusions of this article will be made available by the authors, without undue reservation.

## Author Contributions

RT: primary author and collator of data. AK: coauthor and contributed to data collection, body of manuscript, editing, and proof reading. KB: coauthor and contributed to data collection, body of manuscript, editing, and proof reading. GM: laboratory technician and contributed to Euroflow reporting and methodology. SF: coauthor and contributed to Euroflow reporting and methodology. AS: coauthor and supervisor and contributed to data collection, body of manuscript, editing, and proof reading. All authors listed have made a substantial, direct, and intellectual contribution to the work and approved it for publication.

## Funding

This study received funding from the Department of Haematology, Alfred Health.

## Conflict of Interest

AK has received honoraria from Amgen, Celgene, Pfizer, Roche, CSL, and Sandoz. KB has received consultancy fees from Celgene and travel funding from Amgen. AS has received honoraria and research funding from Celgene, Janssen-Cilag, Amgen, and Takeda; honoraria from STA; and research funding from BMS.

The remaining authors declare that the research was conducted in the absence of any commercial or financial relationships that could be construed as a potential conflict of interest.

## Publisher’s Note

All claims expressed in this article are solely those of the authors and do not necessarily represent those of their affiliated organizations, or those of the publisher, the editors and the reviewers. Any product that may be evaluated in this article, or claim that may be made by its manufacturer, is not guaranteed or endorsed by the publisher.
